# Diffuse intrinsic pontine glioma: current insights and future directions

**DOI:** 10.1186/s41016-020-00218-w

**Published:** 2021-01-11

**Authors:** Dilakshan Srikanthan, Michael S. Taccone, Randy Van Ommeren, Joji Ishida, Stacey L. Krumholtz, James T. Rutka

**Affiliations:** 1 Cell Biology Program, The Hospital for Sick Children, 686 Bay St, Toronto, ON M5G 0A4 Canada; 2 The Arthur and Sonia Labatt Brain Tumour Research Centre, The Hospital for Sick Children, 686 Bay St, Toronto, ON M5G 0A4 Canada; 3 Department of Laboratory Medicine and Pathobiology, University of Toronto, Toronto, ON Canada; 4 Division of Neurosurgery, Department of Surgery, The Ottawa Hospital, Ottawa, ON Canada; 5 Developmental and Stem Cell Biology Program, The Hospital for Sick Children, 686 Bay St, Toronto, ON M5G 0A4 Canada; 6 Institute of Medical Sciences, University of Toronto, Toronto, ON Canada; 7 Division of Neurosurgery, Department of Surgery, The Hospital for Sick Children, Suite 1503, 555, University Avenue, Toronto, ON M5G 1X8 Canada

**Keywords:** Diffuse intrinsic pontine glioma, Molecular genetics, Neuro-oncology, Therapeutics, Disease models, Pediatrics, Neurosurgery

## Abstract

Diffuse intrinsic pontine glioma (DIPG) is a lethal pediatric brain tumor and the leading cause of brain tumor–related death in children. As several clinical trials over the past few decades have led to no significant improvements in outcome, the current standard of care remains fractionated focal radiation. Due to the recent increase in stereotactic biopsies, tumor tissue availabilities have enabled our advancement of the genomic and molecular characterization of this lethal cancer. Several groups have identified key histone gene mutations, genetic drivers, and methylation changes in DIPG, providing us with new insights into DIPG tumorigenesis. Subsequently, there has been increased development of in vitro and in vivo models of DIPG which have the capacity to unveil novel therapies and strategies for drug delivery. This review outlines the clinical characteristics, genetic landscape, models, and current treatments and hopes to shed light on novel therapeutic avenues and challenges that remain.

## Background

Diffuse intrinsic pontine glioma (DIPG) is a lethal malignant pediatric tumor that grows diffusely in the pons. This devastating disease has a median age at diagnosis of 6–7 years and is seldom identified in adults. Given its eloquent location, current treatment options are limited and prognosis is dismal—with less than 10% of patients surviving beyond 2 years from the time of diagnosis [1]. DIPGs represent 80% of all pediatric brain tumors that occur in the brainstem [2, 3]. Histologically, these tumors share features with anaplastic astrocytomas (grade III) or glioblastomas (GBM) (grade IV) [4]. Under the World Health Organization 2016 classification of brain tumors, pediatric gliomas with a K27M mutation in histone H3 (3.1 or 3.3) with a diffuse growth pattern in a midline location are termed diffuse midline glioma, H3 K27M mutant; this designation is inclusive of DIPG cases bearing the K27M mutation [4].

In recent years, significant advancements have been made in our understanding of the molecular underpinnings of these tumors. Previously, pediatric high-grade gliomas (HGG) were thought to resemble adult HGG and were clinically managed as such [5]. However, it is now appreciated that distinct molecular alterations distinguish DIPG from their adult HGG counterparts [6]. This review will present a brief overview of DIPG including its presentation, classification, and treatment options. We also summarize current research and future directions.

## Clinical presentation and diagnostic considerations

Patients with DIPG can present with a wide variety of neurological symptoms reflective of the anatomic localization of the lesion. Thus, in over 50% of patients, cranial nerve palsies (facial asymmetry and diplopia), long tract signs (hyperreflexia, upgoing Babinski), and cerebellar signs (ataxia, dysmetria) are present [7, 8]. Together, these three frequently occurring clinical characteristics are referred to as the “classic triad” and should raise clinical suspicion of this diagnosis prompting appropriate diagnostic imaging. Given that DIPG progresses rapidly, children typically manifest symptoms for a month or less before coming to clinical attention [9]. Cranial nerves VI and VII are the most commonly affected and specific dysfunction of these is characteristic of DIPG [8]. Furthermore, while obstructive hydrocephalus with elevated intracranial pressure is observed in less than 10% of patients at the time of diagnosis, the condition is commonly noted in patients reaching the end-stage of their disease [10].

**Fig. 1:**
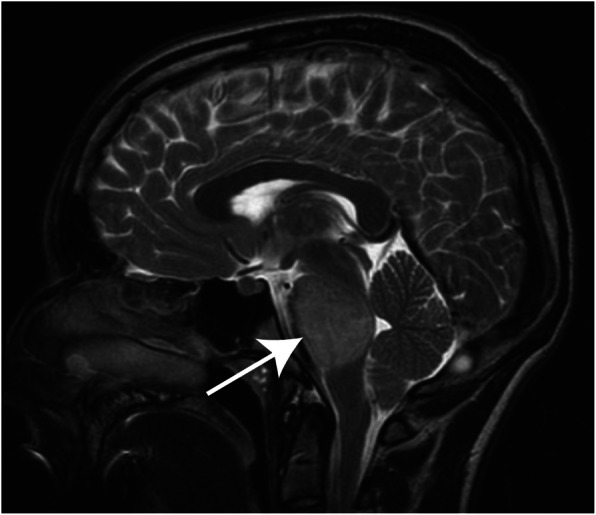
T2-weighted sagittal pediatric brain MRI. Characteristic diagnostic T2-weighted MRI of pediatric DIPG. Note brainstem which demonstrates a diffuse expansile hyperintense lesion in the pons (arrow). These tumors are surgically unresectable due to their poorly circumscribed border and highly eloquent location. Where safe-entry zones are obeyed and neuromonitoring is employed, incisional biopsies under direct observation can be performed

Classically the diagnosis of DIPG has been made based on clinical presentation and neuroimaging findings alone. Magnetic resonance imaging (MRI) is the imaging modality of choice for diagnosis, though on occasion computed tomography (CT) may also be used [11]. Due to the infiltrative nature of these tumors, DIPGs demonstrate T1-hypointensity with ill-defined margins and hyperintensity on T2 weighted images—generally without contrast enhancement (Fig. 1) [8, 12]. Gadolinium enhancement may be of value in confirming the diagnosis or in ruling out other lesions. On imaging, the tumor core is centered in the pons and at the time of presentation can occupy greater than 50% of its axial diameter, typically engulfing the basilar artery. Although DIPGs grow infiltratively and diffusely along fiber tracts to adjacent locations such as the thalamus and cerebellum, they rarely metastasize to distant sites [13].

Historically, advances in imaging techniques rendered biopsies unnecessary to establish a diagnosis in the case of typically presenting DIPGs [14]. This, combined with the perceived risk of obtaining tumor tissue from the eloquent brainstem, has resulted in a relative lack of availability of DIPG samples worldwide to support basic science research. However, with the growing role of molecular diagnostics in DIPG, the relevance and safety of brainstem biopsies have helped move the field forward [15, 16]. A number of studies have shown that biopsies can be performed safely [17–19] and many centers have begun to use stereotactic biopsy as standard practice in an effort to enhance diagnosis and support basic science research and as a gateway for entry into ongoing clinical trials which require histological and molecular data [15, 16, 20, 21]. Our own tertiary pediatric care center also advocates for this approach. Although currently limited, the acquisition of tumor tissue which yields insights into the molecular phenotypes of this heterogeneous disease will provide physicians with significant insights into diagnosis, treatment, and prognosis of children.

**Fig. 2:**
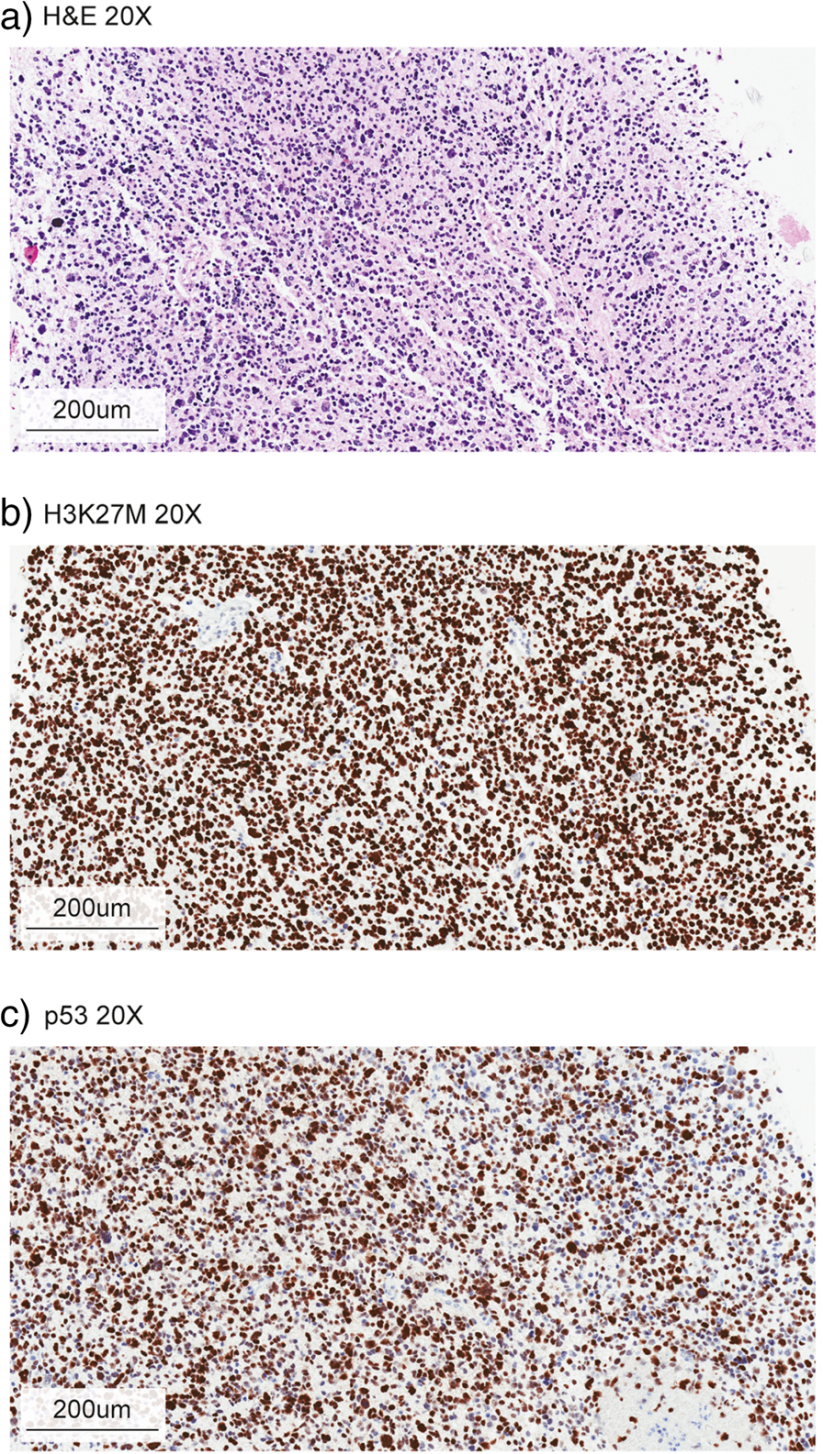
Representative histology of pediatric DIPG. **a** Hematoxylin & eosin stain of pediatric DIPG acquired post-mortem, note diffusely infiltrative tumor cells amidst a background matrix of neuropil. **b** Immunohistochemical staining of the same sample using antibody directed to the H3K27M epitope. The sample is strongly positive. **c** Immunohistochemical staining of mutant p53, a common co-occurring mutation in DIPG. Note sparser staining than H3K27M in panel **b**

Differential diagnostic considerations include non-malignant brainstem entities including low-grade glioma, primitive neuroectodermal tumor (PNET), vascular malformations, encephalitic parenchymal lesions, cysts, and demyelinating disorders [22, 23]. When tissue is available through biopsy, the diagnosis can be confirmed by histological review, supplemented by molecular testing where available. Microscopically, cases often show high-grade astrocytic histology, with findings of increased mitotic activity, microvascular proliferation, and/or necrosis (Fig. 2) [10]. A smaller percentage of cases however will show lower grade histology with overall bland cytology lacking some or all the traditional high-grade features. In addition to typical glioma immunohistochemistry panels such as GFAP, ATRX, p53, neurofilament, ki-67 immunostains, targeted antibodies for H3K27M, BRAF-V600E, and IDH1-R132H may be applied (Fig. 2 b and c). Various molecular pathology approaches including next-generation sequencing and DNA microarrays are utilized to molecularly confirm the presence or absence of a histone 3 (H3) mutation and identify the histone isoform affected given their prognostic differences. Although therapeutically relevant mutations such as BRAF-V600E are quite rare in DIPG tumors [24], evaluation is generally attempted given the availability of targeted therapies such as dabrafenib or vemurafenib [25]. A useful summary of clinicopathologic features of DIPG can be found in Table 1.

**Table 1:**
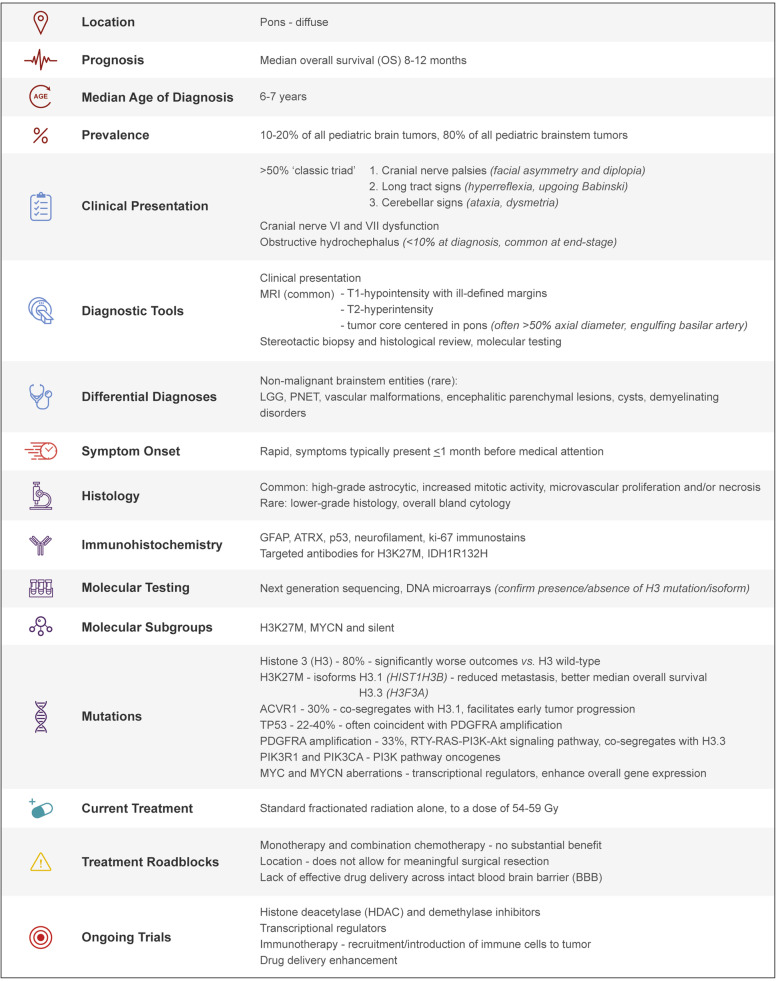
Summary of our current knowledge of pediatric DIPG. Summary table which details key clinical, pathological, and genetic features of pediatric diffuse intrinsic pontine glioma. *LGG*: low-grade glioma; *PNET*: primitive neuroectodermal tumor

## Molecular characteristics and subgroups

DIPGs can be sub-classified into 3 distinct molecularly defined groups: H3K27M, MYCN, and silent [26]. Previous research has identified that while DIPGs do share similarities with supratentorial HGG, it remains a unique entity with distinct genomic and molecular alterations [27]. For example, H3 mutations are identified in nearly 80% of DIPGs whereas only 35% of pediatric non-brainstem high-grade gliomas have H3 mutations [27]. Although many hallmark mutations have been identified in DIPG, it is important to note that intra- and inter-tumoral heterogeneity has been documented in this disease [28].

Histone mutations are present in the majority of DIPG tumors, and the identification of these mutations has resulted in a paradigm shift that has redefined our focus of research and clinical management [29, 30]. The histone mutation H3K27M results in the substitution of lysine with methionine in the isoforms H3.1 and H3.3, encoded by genes *HIST1H3B* and *H3F3A* respectively. This mutation leads to the loss of histone trimethylation via inhibition of polycomb repressive complex 2 (PRC2), ultimately producing epigenetic silencing [29, 31]. However, despite extensive modeling using in vivo mouse models, the precise role of H3K27M in tumor initiation remains elusive [32].

There are subtle differences between the histone mutations in H3.1 and H3.3, particularly regarding survival, phenotype, and clinical outcomes [30, 31]. H3.1 histone mutations tend to be associated with slightly improved survival and reduced presence of metastasis [33]. Overall, in comparison with other H3 wild-type cases, the H3K27M is associated with significantly worse outcomes irrespective of the isoform affected [34].

ACVR1 mutations have been identified in approximately 30% of DIPG tumors, and co-segregate with H3.1 [26, 27, 35]. It has been previously shown that *ACVR1* mutation facilitates early tumor progression coupled with other molecular aberrations, and shows promise for therapeutic targeting [36]. *TP53* mutations have been identified in approximately 22–40% of DIPGs and often co-occur with PDGFR amplification [37, 38]. *TP53* mutation, coupled with H3.3K27M and typically *PPM1D* mutations have been demonstrated to allow tumor cells to evade cell death and senescence by influencing epigenetic regulation [39].

*PDGFRA* is the most commonly observed amplification, present in approximately one-third of high-grade gliomas and is implicated in the RTK-RAS-PI3K-Akt signaling pathway [40]. *PDGFRA* causes activation of PI3K and MAPK pathways via phosphorylation at various phosphotyrosine domains [40]. Amplifications of *PDGFRA* co-segregate with histone H3.3 mutations and are clinically aggressive regardless of histological classifications [30, 31, 38].

In addition to *PDGFRA*, *PIK3R1* and *PIK3CA* are also drivers of the PI3K pathway and have been found to contribute to an aggressive phenotype in DIPG [28, 37]. These mutations have been characterized as an obligatory partner in H3.3K27M and are reported in clonal populations of DIPG. Lastly, MYC and MYCN aberrations are present in DIPGs and act as transcriptional regulators that enhance gene expression genome-wide [27]. Further investigation of the genomic landscape and the biological underpinnings of this disease is prudent to characterize important oncogenic drivers/pathways and subsequent actionable targets [27]

## Treatment challenges and obstacles to progress

The current standard of care for DIPGs consists of standard fractionated radiation alone to a dose of 54–59 Gy, as any chances of meaningful surgical resection are limited by the eloquent location of DIPGs [41]. Furthermore, many treatment regimens, including monotherapy and combination chemotherapies have thus far yielded no substantial benefit [5, 42, 43]. Recent advances in the field of immunotherapy however have identified a potential role for anti-GD2 chimeric antigen receptor (CAR) T-cell therapy, which may show potential efficacy [44]. These limited treatment options highlight the need for novel therapeutic approaches. Herein we describe possible targets and common obstacles to effective therapies.

A variety of oncogenic drivers and somatic mutations in DIPG contribute to its rapid tumorigenesis and dismal outcomes. As previously mentioned, the most common mutation involves the substitution of a lysine for methionine at position 27 in histone H3, particularly in histone 3.1 and 3.3, which is associated with a worse prognosis over their wild-type counterparts [30, 45, 46]. DIPGs tend to have either a somatic mutation in H3K27M and/or a global loss of H3K27 trimethylation; as such, this is suggested to be one of the oncogenic drivers of this disease [31]. The presence of H3K27M leads to various downstream chromatin remodeling cascades, epigenetic silencing, and activation of various genes and pathways [47, 48]. The identification of this mutation and discoveries of subsequent secondary mutations open the door to druggable targets such as histone deacetylase (HDAC) and demethylase inhibitors—some of which have shown promising results [34, 49, 50]. Studies have also found that targeting transcriptional regulators via activation of bromodomain proteins has been effective in preclinical models [49, 51]. Although many DIPGs occur with the histone mutation, many targetable secondary mutations have also been identified that play a role in tumorigenesis [26, 35, 45, 52].

Previously, one of the most common obstacles regarding DIPG research and target identification was the lack of available tumor tissue. However, with increasing acquisition of post-mortem tissues and biopsies, several molecular studies can now be performed robustly and reproducibly. As a result, many promising targets have been identified and several drugs have shown efficacy in the preclinical setting. However, there remains a considerable obstacle between clinical application and drug discovery due to the lack of effective drug delivery across an intact blood-brain barrier (BBB). This may also explain why drugs that show efficacy in other gliomas have failed in DIPG [53]. Improving drug delivery, as a result of structural adaptation or physical disruption of the BBB will be vital for novel therapies to be translated into the clinic.

Lastly, the tumor microenvironment is a critical component of the tumor to consider when deciding treatment, particularly immunotherapy. Recent studies have concluded that DIPGs possess a non-inflammatory tumor microenvironment [54, 55]. However, whether DIPG tumors contain tumor-associated macrophages has yet to be fully investigated as there are conflicting results that state DIPGs do not have increased macrophage infiltration [54] or that DIPGs have increased macrophage infiltration but do not secrete inflammatory cytokines [55]. That said, most studies demonstrate that there is no T-cell infiltration in DIPG, and thus immunotherapeutic approaches should be focused on the recruitment or introduction of immune cells to the tumor.

## Experimental models of DIPGs

The rare occurrence and eloquent location of DIPG make it difficult to obtain comprehensive tumor tissue that accurately reflects the intratumoral heterogeneity of this disease. Thus, more so than in other cancers, the establishment of biologically representative models is critical in revealing its genomic and epigenomic underpinnings. Patient-derived cell lines from biopsy and post-mortem tissue have allowed experiments in vitro that elucidate many targets and functional pathways [56]. Most groups have either utilized neurosphere [34, 57–59] or adherent monolayer patient-derived cell cultures [53, 60–62] for in vitro testing of novel drugs and targets. Historically, glioma cells have been cultured as adherent monolayers in the presence of fetal bovine serum (FBS); recently, however, 3-dimensional serum-free culture methods have become increasingly popular for in vitro drug testing. Moreover, recent research has demonstrated that culture conditions including culture media components and oxygen concentrations in the culture environment can cause major changes in gene expression, pathway activation and subsequently influence the validity of in vitro response to therapies [63, 64].

The first attempts to develop animal models of DIPG involved intracranial injections of rat glioma cell lines into the brainstem of neonatal and adult rats in order to recapitulate brainstem gliomas [65–68]. Although these models did faithfully produce tumors resembling gliomas in the appropriate location, one major criticism of this approach is that the tumor cells are derived from gliomas that arose in the cerebral cortex which biologically differ from gliomas which arise in the brainstem [10]. Several groups have also generated xenograft models either from human adult hemispheric GBM derived cells or from established GBM cell lines by serial transplantation of xenografts into the brainstem of immunodeficient rats or mice [69–71]. However, the caveat with this model is that the use of glioma cells from the cerebrum, which regardless of growing in the brainstem microenvironment, may not adequately recapitulate DIPG. In an effort to address the challenges mentioned, Monje and colleagues were the first to develop DIPG-specific cell and xenograft lines from post-mortem tissue [39]. Since then, tissues harvested from living biopsies are beginning to emerge, as groups have developed DIPG cell lines from tumor samples harvested at diagnosis [62, 72].

In addition to human xenograft mouse models, genetically engineered mouse models (GEMMs) have proven useful for the elucidation of genetic alterations, oncogenic drivers, and lineages of clones in tumor cells in immune-competent models [73]. These GEMMs have the advantage of being immune competent and are therefore useful for pre-clinical trials of immunotherapeutic approaches. Furthermore, they are far more accurate in recapitulating the tumor microenvironment in comparison to xenograft models [74]. Earlier GEMMs were generated using the replication-competent avian sarcoma-leucosis virus (RCAS) vector to enable Ink4a-ARF loss and platelet-derived growth factor B (PDGFB) overexpression within nestin-expressing cells in the pons of genetically engineered pups expressing tumor virus A (TVA) under the nestin promoter [75, 76]. Although, these attempts created successful infiltrative tumors, they were not exclusive to the pons [77, 78]. More recent models of DIPG GEMMs have utilized specific genetic alterations such as *PDGFB*, *H3K27M*, and *p53* [79] although these models are also not exclusive to the pons*.* Recently, models of *ACVR1* have elucidated that mutant *ACVR1* arrests glial cell differentiation and subsequently drives tumorigenesis in pediatric gliomas [80]. In the future, the development of faithful genetic models of DIPGs that consistently recapitulate both spatiotemporal and molecular tumor characteristics will be vital for identifying novel oncogenic programs and elucidating the temporal and spatial factors which contribute to tumor formation.

## Novel therapeutic avenues

There are multiple therapeutic avenues for DIPG that hold promise for the future. These include targeted therapies, epigenetic therapy, and immunotherapy. Here, we briefly describe each of these avenues and highlight their current state of development and their significance.

Since the development of targeted therapies for DIPG, approximately 250 clinical trials have been initiated against different biological pathways in the disease [81]. One of the most frequently amplified genes is *PDGFRA* which is found in 10% of DIPGs. As a result, *PDGFRA* is one of the most targeted genes for therapy in DIPG [38, 82]. However, agents that target PDGFR such as imatinib and dasatinib have exhibited fairly poor antitumor effects in clinical trials [62]. Another gene that has been targeted in DIPG is *EGFR*, which has also been shown to be overexpressed in pediatric brain tumors [83]. Clinical trials of anti-EGFR drugs including nimotuzumab, gefitinib, and erlotinib have shown some benefit in small subsets of DIPG patients [84–86]. Other trials have used PARP1 inhibitors (olaparib, niraparib, veliparib), CDK4/CDK6 inhibitors (PD-0332991), WEE1 kinase inhibitor (MK1775), and the angiogenesis inhibitor (bevacizumab) [87–89]. Despite various clinical trial attempts, none of these has shown significant efficacy in DIPG. One of the rate-limiting steps in these clinical trials is incomplete knowledge of whether these agents cross the BBB [53].

**Fig. 3:**
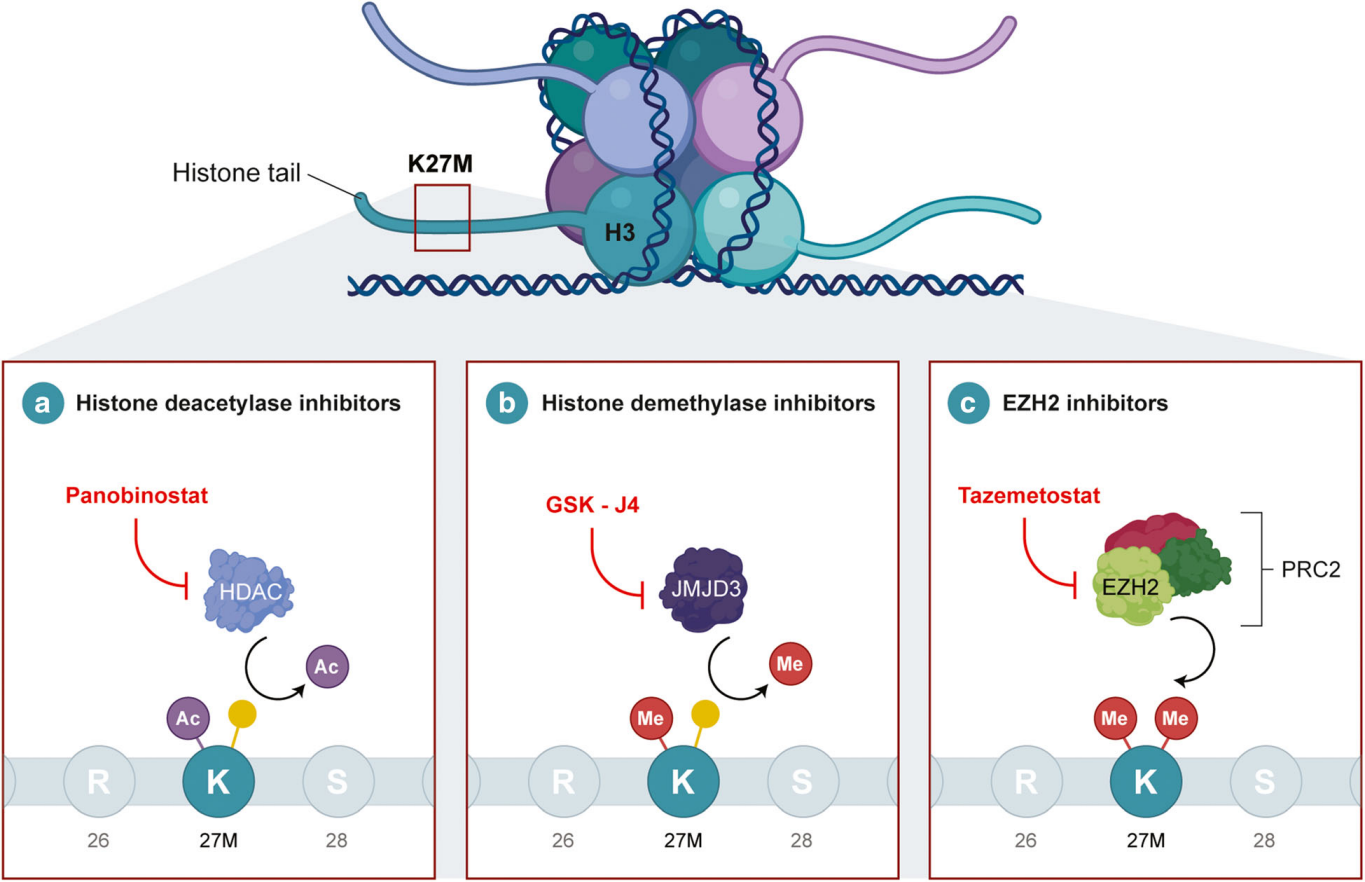
Drug targets and treatments of pediatric DIPG. DIPGs are characterized by the K27M mutation that occurs in histone H3. Therapies which target the epigenome including histone deacetylase inhibitors such as panobinostat (**a**) and histone demethylase inhibitors such as GSK-J4 (**b**) have been demonstrated to be effective in clinical trials. Furthermore, residual PRC2 activity has been shown to be required for proliferation and thus EZH2 inhibitors such as tazemetostat (**c**) has been effective in treating these tumors

**Fig. 4:**
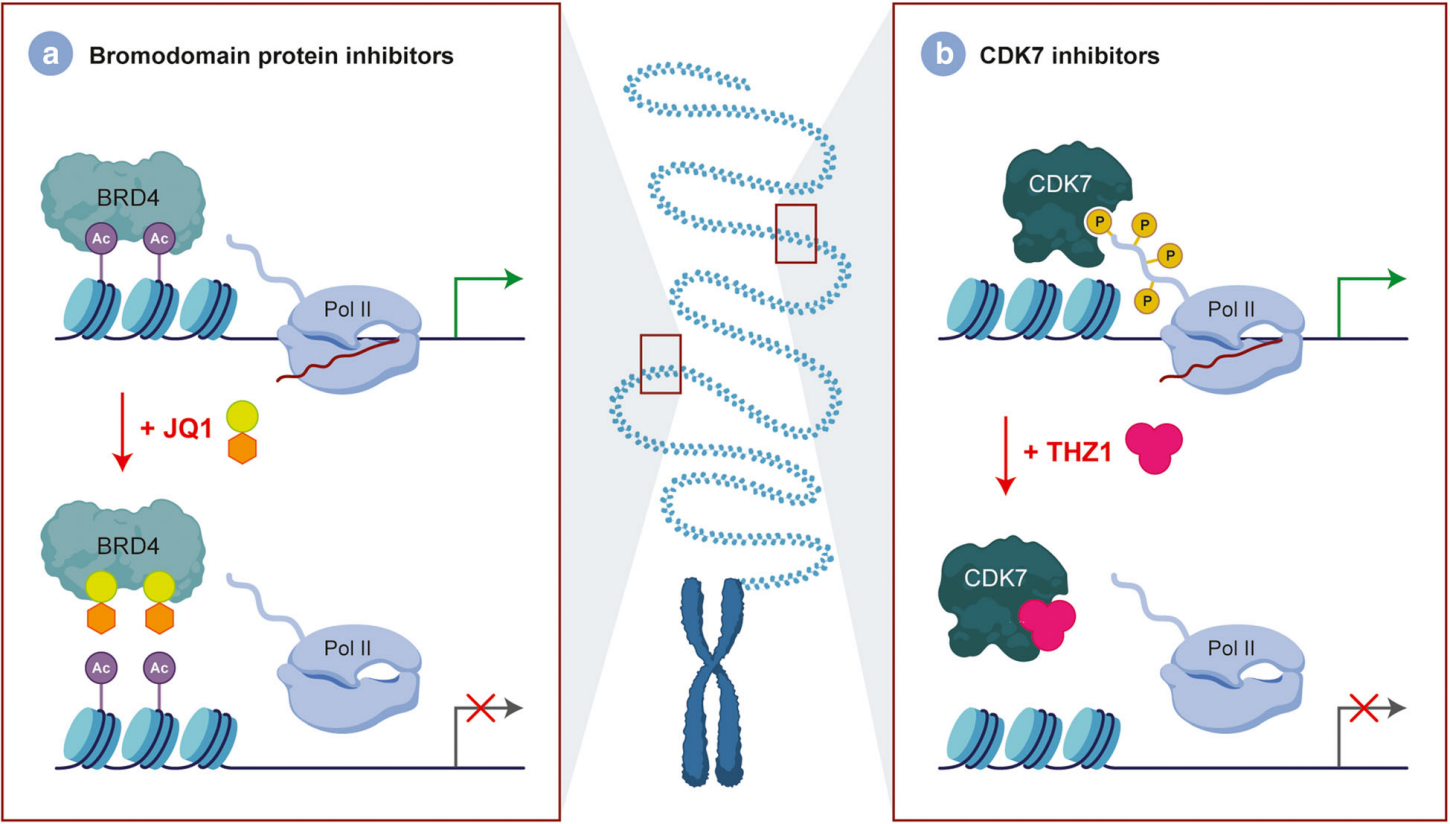
Targetable transcriptional dependencies in DIPGs. DIPGs can also be targeted pharmacologically at the DNA level by leveraging the transcriptional dependencies of the tumor. Transcriptional disruption has been demonstrated to reduce tumor growth via bromodomain (**a**) or CDK7 (**b**) inhibition using JQ1 or THZ1, respectively

Recently, substantial evidence has suggested that epigenetic alterations, coupled with genetic mutations, are responsible for tumorigenesis. Studies using JMJD3 inhibitors such as panobinostat and GSK-4, which target histone deacetylase and demethylase, respectively, have shown promising results and at present have moved into clinical trials as both single and combination agents (Fig. 3 a and b) [34, 90, 91]. The decreased H3K27me3 levels have led to unique strategies that target chromatin remodelers. Enhancer of zeste homolog 2 (EZH2) is a H3K27-methylating enzyme and was found to be highly expressed in H3K27M-mutant DIPG [92]. However, treatment with the EZH2 inhibitor, EPZ6438, has yielded little to no results in both GBM and DIPG cell lines [93]. In contrast, tazemetostat (Fig. 3c) which is also an EZH2 inhibitor has yielded significantly better results, although this may be due to sample selection bias [94]. On the other hand, studies have also targeted enzymes responsible for demethylation of H3K27 such as JMJD3 [95]. Transcriptional regulators such as BET family proteins have also been investigated as targeted for therapy in brain tumors. JQ1 has been found to be a histone-binding module inhibitor that binds to the bromodomains and displaces the BRD4 fusion oncoproteins subsequently leading to cell cycle arrest and apoptosis (Fig. 4a) [96]. In addition, alternatives methods of disrupting transcription have also been effective such as CDK7 inhibition with THZ1 (Fig. 4b) [51]. Apart from these molecular targets, several other secondary mutations have yet to be investigated as therapeutically actionable (Table 2).

**Table 2:**
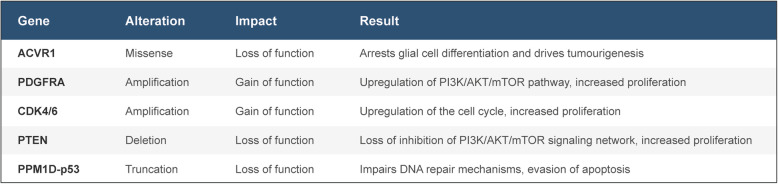
Potential targetable secondary mutations in pediatric DIPG. Summary table which outlines key secondary genes that are altered in DIPGs and their subsequent result which increases tumorigenesis. We suggest that these secondary mutations can be complimentarily targeted in order to effectively treat DIPGs

Emerging evidence has linked epigenetics and metabolomics to plasticity and intratumor heterogeneity in brain tumors [97–100]. Specifically in DIPGs, recent studies have found that metabolic reprogramming contributes to the pathogenesis of H3.3K27M DIPGs, primarily by utilizing alpha-ketoglutarate to maintain a preferred epigenetic state of low H3K27me3 [97]. Furthermore, they also show that H3.3K27M cells show intratumoral heterogeneity in their usage of glucose or glutamine to regulate global H3K27me3 with dependence on one or both pathways [97]. In return, the metabolic regulation of global H3K27me3 leads to heterogeneous dependencies on glutamate dehydrogenase, hexokinase 2, and wild-type isocitrate dehydrogenase 1 (IDH1), which are possible therapeutic targets. Leveraging these metabolic and epigenetic vulnerabilities in DIPG should be a priority for future treatment strategies.

Immunotherapy is rapidly establishing itself as a pillar of cancer therapy, and recent studies have demonstrated its potential in brain tumors [101–105]. In particular, an anti-GD2 CAR-T study portrayed positive results in DIPG in vivo models [44]. However, it is important to note that the administration of these GD2 CARs had resulted in hydrocephalus that was lethal in a subset of animals which was suggested to be due to the neuroanatomical location of the tumors to the cerebrospinal fluid (CSF) pathways [44]. Given these challenges, the need for identification of novel strategies in administrating immunotherapy will be required to advance this promising treatment into clinical trials.

## Conclusion

The elaborate molecular pathogenesis, strict BBB regulation, and eloquent location have contributed to the current lack of improvements in prognosis for DIPG. Radiation therapy remains the mainstay of care. However, with an increasing understanding of its molecular genetics, a growing number of promising preclinical models, and novel techniques to overcome the limitations of effective drug delivery across the BBB, it is our hope that the future of DIPG therapy will change dramatically in a relatively short time, much to the benefit of children who harbor this devastating brain tumor.

## Data Availability

Not applicable

## Abbreviations

DIPG: Diffuse intrinsic pontine glioma
GBM: Glioblastoma
WHO: World Health Organization
HGG: High-grade glioma
MRI: Magnetic resonance imaging
CT: Computed tomography
PNET: Primitive neuroectodermal tumor
GFAP: Glial fibrillary acidic protein
PRC2: Polycomb repressive complex 2
ACVR1: Activin A receptor, type I
PDGFR: Platelet-derived growth factor receptor
PPM1D: Protein phosphatase, Mg^2+^/Mn^2+^-dependent 1D
MAPK: Mitogen-activated protein kinase
CAR: Chimeric antigen receptor
PI3K: Phosphoinositide 3-kinases
HDAC: Histone deacetylase
FBS: Fetal bovine serum
BBB: Blood-brain barrier
GEMM: Genetically engineered mouse models
RCAS: Replication-competent avian sarcoma-leukosis virus
TVA: Tumor virus A
PDGFB: Platelet-derived growth factor B
PDGFRA: Platelet-derived growth factor receptor A
EZH2: Enhancer of zeste 2
EGFR: Epidermal growth factor receptor
CDK4/6: Cyclin-dependent kinase 4/6
PARP1: Poly(ADP-ribose) polymerase 1
MJD3: Jumonji domain-containing protein D3
BRD4: Bromodomain-containing protein 4
CSF: Cerebrospinal fluid
IDH1: Isocitrate dehydrogenase 1
